# Sensitivity and accuracy of high-throughput metabarcoding methods for early detection of invasive fish species

**DOI:** 10.1038/srep46393

**Published:** 2017-04-13

**Authors:** Chelsea Hatzenbuhler, John R. Kelly, John Martinson, Sara Okum, Erik Pilgrim

**Affiliations:** 1 Mid-Continent Ecology Division, National Health and Environmental Effects Research Laboratory, Office of Research and Development, Environmental Protection Agency, Duluth, MN, 55804, United States of America; 2 Oak Ridge Institute for Science and Education Participant, Oak Ridge, TN, 37831, United States of America; 3 Exposure Methods and Measurements Division, National Exposure Research Laboratory, Office of Research and Development, Environmental Protection Agency, Cincinnati, OH, 45268, United States of America; 4 Systems Exposure Division, National Exposure Research Laboratory, Office of Research and Development, Environmental Protection Agency, Cincinnati, OH, 45268, United States of America

## Abstract

High-throughput DNA metabarcoding has gained recognition as a potentially powerful tool for biomonitoring, including early detection of aquatic invasive species (AIS). DNA based techniques are advancing, but our understanding of the limits to detection for metabarcoding complex samples is inadequate. For detecting AIS at an early stage of invasion when the species is rare, accuracy at low detection limits is key. To evaluate the utility of metabarcoding in future fish community monitoring programs, we conducted several experiments to determine the sensitivity and accuracy of routine metabarcoding methods. Experimental mixes used larval fish tissue from multiple “common” species spiked with varying proportions of tissue from an additional “rare” species. Pyrosequencing of genetic marker, COI (cytochrome *c* oxidase subunit I) and subsequent sequence data analysis provided experimental evidence of low-level detection of the target “rare” species at biomass percentages as low as 0.02% of total sample biomass. Limits to detection varied interspecifically and were susceptible to amplification bias. Moreover, results showed some data processing methods can skew sequence-based biodiversity measurements from corresponding relative biomass abundances and increase false absences. We suggest caution in interpreting presence/absence and relative abundance in larval fish assemblages until metabarcoding methods are optimized for accuracy and precision.

Aquatic invasive species (AIS) endanger the economic and ecological constitution of successfully colonized natural ecosystems. Ecological impacts resulting from increased predation^1^, parasitism^2^, interspecific competition^1^,^3^, or habitat disturbance^4^,^5^ associated with unchecked AIS populations often have negative economic consequences such as reduced native commercial sport and forage fish populations^1^,^2^,^6^,^7^ or industrial and recreational water use impairments accompanying AIS biofouling^8^,^9^. Despite extensive management efforts focused on preventing new introductions and controlling the spread of established populations AIS remain an enduring threat to many aquatic communities^10^,^11^. The continued spread of AIS has encouraged development of AIS early detection methods targeting invasion prone locations to detect new invaders during early stages of the invasion process when individuals are present at low abundance (rare) and the population is localized^12^,^13^. Nonetheless, “rare” can be hard to find and requirements for an adequate search can be costly. Moreover, detection errors can occur during sample collection or taxonomic identification in the field or lab and failing to detect a newly introduced species restricts our ability to manage burgeoning populations.

A practical early detection strategy balances the search effort with an acceptable amount of non-detection risk^14^,^15^ for a given detection probability. Developing a practical strategy involves quantifying detection limits and error related to the search and species identification/validation methods, as well as optimizing the entire process to increase detection efficiency. Typically, adult populations are monitored for early detection of invasive fish species^14^, but monitoring larval fish communities may provide some benefits over adult surveillance because detecting new invaders in larval form would more strongly suggest the presence of a successfully reproducing population posing an imminent threat. Moreover, detection efficiencies may be gained through sampling the larval life stage because larval fish are more abundant, may occupy different habitats, and may be less likely to avoid capture than their adult counterparts^15^,^16^. Traditional taxonomic identification of larval fishes, however, presents significant challenges for a practical and successful early detection program. The morphological ambiguities at the larval life stage impede the accurate, high-resolution classifications (i.e., species level)^17^,^18^,^19^,^20^,^21^,^22^ required to maintain a low probability of detection errors. Furthermore, sample processing and identification of numerous individuals creates a substantial delay between sample collection and completion of identifications which has major ramifications for the “early detection” concept^15^,^23^,^24^. Due to the challenges associated with traditional taxonomic identification of larval fishes, monitoring larval fish communities for early detection of invasive fish species may only be practical if an alternative identification method is employed.

Advancements in molecular genetics diagnostics hold promise as an alternative to traditional morphological taxonomy in an AIS early detection strategy. High-throughput sequencing (HTS), or metabarcoding, enables simultaneous sequencing of a high-resolution genetic marker (DNA barcode) in many samples (multiplexing) providing a fast, and potentially cost-effective method for estimating biodiversity in multi-species assemblages^25^,^26^,^27^. Moreover, instrument sensitivity assessements conducted with marine and aquatic invertebrates^28^,^29^,^30^ demonstrated HTS provides a means to accurately describe species richness, and the lowest limit of detection, for tested invertebrate communities, is very sensitive. Nonetheless, sample composition (e.g., life stage, relative abundances) can vary greatly within and between samples, which may influence the limits to detection. Furthermore, the HTS workflow is complex, comprising many factors that can influence detectability. Sample collection and processing methods affect the quality of DNA extracted from the samples. Genetic marker selection, PCR design, and downstream sequence data processing methods influence taxonomic resolution and accuracy of the final biodiversity estimates^31^,^32^,^33^,^34^,^35^. For example, data processing methods used to remove low quality and potentially erroneous, or biologically irrelevant sequences from final sequence biodiversity estimates^36^ can exclude genetic signals represented by very few sequences (weak signals) despite biological relevance^35^. Weak signals may correspond to a low abundance of starting material or ensue from differential barcode amplification (PCR bias) that can skew sequence biodiversity estimates from corresponding relative biomass abundances^32^,^33^. Extreme biases may increase non-detection risk for under-represented or rare taxa.

Although development of DNA based detection methods is progressing, our understanding of the limits to detection for metabarcoding complex samples is inadequate. Consequently the utility of high-throughput metabarcoding methods for AIS management, namely early detection monitoring remains in question. Using larval fish as a relevant life stage, we carried out several experiments designed to investigate the sensitivity and accuracy of metabarcoding methods commonly used to characterize composition of samples with a mixture of species from the larval fish community.

## Methods

### Experimental design

Multi-species assemblages were constructed using whole tissue or biomass from larval fish categorized as “non-target” or “target” species to represent a common or rare presence, respectively. Species were primarily selected based on the availability of biomass in the field-collected samples from which we sourced our tissue for constructed samples. To limit sample matrix complexity selected species were from distinct families or genera, using specimens similar in size or developmental stage. A preliminary experiment was designed to define workflow processes that influence detectabilty. Preliminary results directed design and method modifications to improve detectability in the second experiment that aimed to assess detection limits in samples with differing degrees of species richness. To evaluate instrument sensitivity and accuracy, sequence biodiversity was compared to corresponding biomass relative abundances. Each design comprised three sample types, i) single species control, ii) Treatment 1 (T1) a proportionate mix of non-target biomass, target excluded, and iii) a suite of test mixes (Treatments 2–7; T2–T7). In T2–T7 non-target matrices with 1:1 biomass ratios between taxa were spiked with varying proportions of biomass from an additional target species, the percentage of target biomass in each treatment reflected the probability of detecting the target species (e.g., target biomass is 1% of total sample mass, so theoretical probability of detection is 1 in 100).

### Trial A

The target selected for our preliminary experiment (Trial A) was *Proterorhinus semilunaris* (target A) and four species constituted the non-target tissue mix in each treatment (Table 1). Species richness (S = non-target + target taxa, S = 5) mirrored average richness observed in natural community samples.

### Trial B

Relative to Trial A, our second experiment (Trial B) was designed to evaluate instrument sensitivity to rare biomass for a restricted range of detection probabilities using a different target species, *Percopsis omiscomaycus* (target B) in three subsets constructed with low (S = 2), intermediate (S = 5) or high (S = 11) species richness (Table 2).

### Larval fish collection and sample construction

Larval fish were collected from the St. Louis River estuary and Duluth-Superior harbor (i.e., Laurentian Great Lakes coastal waters) during June and July 2013. Larval specimens were preserved in 95% non-denatured ethanol at the time of collection then stored at or below 4 °C^37^,^38^,^39^,^40^,^41^. For all laboratory procedures, sample contamination was prevented by wearing sterile, disposable gloves and disinfecting the lab workspace, tools, and glassware between each use. Because of the challenges associated with identifying larval fish, we only selected species for our experiments that we could easily identify to species level with 100% confidence. After fish larvae were identified^42^, specimens from each species were pooled and identifications were verified by a second taxonomist. To limit the potential for measurement error, pipettable tissue homogenates were prepared from each species. Cryogenic grinding with mortar and pestle reduced pooled larvae into small pieces and cryogenic homogenates were desiccated in heat sterilized aluminum weigh pans^39^, weighed, transferred to a known volume of chilled (4 °C) Tris EDTA buffer, pH 8 and rotor-stator homogenized using the polytron stand homogenizer (POLYTRON PT-10735 Homogenizers). Equations used to calculate homogenate concentration and volume are given in Table 3. To construct samples, homogenate aliquots (±0.00076 mg/μL) from each species were pipetted into 2 mL sterile polypropylene tubes and stored at −20 °C until submitted into the sequencing workflow.

### DNA sequencing

Total genomic DNA was extracted according to the manufacturer’s instructions from larval fish mixes using the DNeasy Blood and Tissue kit (Qiagen) and normalized using sterile water to 10 ng template DNA/μL. The genetic marker was a 658 base pair (bp) section of the 5′ end of the mtDNA protein coding gene, cytochrome *c* oxidase subunit I (COI), a standard DNA barcode for identifying fish species^26^,^43^,^44^. COI barcodes were PCR amplified using a universal fish primer cocktail with C_FishF1t1-C_FishR1t1 forward and reverse primers (including M13 tails to facilitate sequencing) at a ratio of 1:1^45^. The PCRs using 20 ng template DNA, 4 μL 1X BSA, 2 μL 10X PCR buffer (Qiagen), 0.6 μL 25 mM MgCl_2,_ 0.4 μL 10X dNTPs, 0.1 μL 10 mM *Taq* DNA polymerase (Qiagen), 0.5 μL of primer cocktail and sterile water for a final volume of 20 μL took place in a Bio-Rad thermocycler, initiated at 94 °C for 150 sec., then 35 cycles of 94 °C for 30 sec., 46 °C for 60 sec., and 72 °C for 60 sec., before a final extension at 72 °C for 10 min. Five replicates were cycled for each sample and pooled prior to PCR product purification with QIAquick PCR Purification Kit (Qiagen). Amplified COI barcodes (amplicons) were quantified and normalized with the same methods used for genomic DNA.

Purified COI amplicons were prepped for pyrosequencing on the Roche GS-FLX+ instrument per manufacturer’s instructions for MID tag multiplexing and amplicon library building, then centrifuged with sequencing reaction enzymes onto a 70 × 75 PicoTiter plate (PTP). Samples in each trial were multiplexed using ten 10 bp MID tags and a multi-region plate gasket. Trial A samples were sequenced on two separate 454 runs placing 18 and 4 samples on 8 and 4 region plates, respectively. In total, 79 samples constructed for Trial B were sequenced on a single run using a 16 region plate. The PTP layouts were designed to provide adequate sequencing depth, meaning the probability of detecting the rare biomass (target species) in a given sample was at least 10X greater than the manufacturer’s lowest estimated number of sequences (reads) per sample (e.g., *P(D*_*t*_) = 1 in 100, est. reads ≥1000).

### Sequence data processing and analysis

The sequence data output was demultiplexed to corresponding treatment/replicate IDs, then MID tags and primers were trimmed from COI barcodes^36^. Concurrent with demultiplexing and primer/tag trimming, sequences were quality filtered based on the quality score (Phred score) assigned to each nucleotide base indicating the accuracy of each base call, a process that determines nucleotide sequences from signal peaks generated during pyrosequencing. A sliding window test of quality scores was used to filter for quality. Sequences were trimmed from the 3′ end to the point where every run of 100 consecutive bases had an average quality score ≥20 (99% accuracy). After trimming, sequences with a total length <200 bases were discarded from further processing^36^. The remaining acceptable sequences were de novo clustered with UCLUST software at ≥97% base similarity into operational taxonomic units (OTUs). The seed (first) sequence of each OTU cluster was selected to represent the cluster^46^. Representative sequences were assigned taxonomy and screened to identify potential chimeric sequences that might have been produced during PCR^36^. Taxonomy was assigned based on a percent match criteria threshold of >90% base similarity to reference sequences. Our reference library database consisted of publicly available COI sequences downloaded from the Barcode of Life Database (BOLD)^47^ augmented by COI voucher sequences obtained from adult fish fin clips from specimens collected from the Laurentian Great Lakes basin and identified by the U.S. EPA Duluth, MN laboratory, the U.S. Fish and Wildlife Service, Ashland WI office, and the Minnesota and Wisconsin Department of Natural Resources.

After assigning taxonomic identities to the unknown COI barcodes, our knowledge of sample composition allowed us to set filtering thresholds to identify and isolate potential false positives resulting from sequencing errors or the presence of small amounts of extracellular DNA shed from fish species present in the bulk tissue samples from which we sourced the fish tissue for constructing our samples. Filtering thresholds were set based on the expected values (the probability of sequence alignment occurring by chance, reflecting the biological relevance of taxonomic assignments) and overall signal strength (number of clustered sequences) of representative OTUs associated with a false presence or low-resolution taxonomic classification (e.g., taxon not used to construct samples, or ‘Perciformes spp.’, respectively). Genetic signals below threshold values were filtered from the data set. The limits to detection were evaluated by comparing final sequence biodiversity estimates to constructed biomass based biodiversity for all replicates. To evaluate how our filtering application affected observed detection limits, data comparisons were made before and after setting filtering thresholds.

## Experimental Results

### Analysis of COI sequence biodiversity in constructed samples (Trial A)

Pyrosequencing of COI markers generated 346,507 sequences on the eight region plate and 371,608 sequences on the four region plate after filtering for quality and removing PCR artifacts from sequence data. For the combined datasets 99.7% of the sequences were assigned to species used to construct the mixes and thus expected in the various treatments. Average percent base similarity of our sequences to reference sequences used to assign taxonomy was 97.2%. Genetic signals for expected species were observed for each single species control and in T1, and T2 replicates constructed with equal biomass proportions between species (Table 1). COI sequences for target A, *P. semilunaris* were recovered in 50% and 25% of replicates with target biomass representing 0.1% (T3) and 0.02% (T5) of total biomass, respectively and positive detection was attributed to 1–5 sequences per hit. Signal for target A was not observed in T4 replicates where target biomass represented 0.04% of the total biomass. COI barcodes for common, non-target species each with an initial biomass ≈ 24.99% were recovered in all replicates constructed for T3–T5.

**Figure 1:**
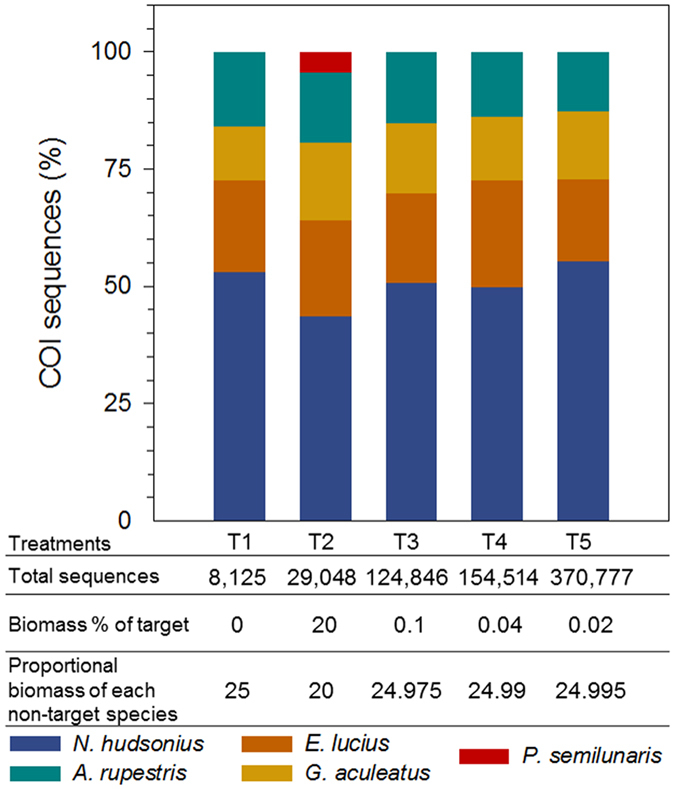
Metabarcoding results from larval fish tissue samples constructed for Trial A before setting false presence filtering thresholds. The observed distribution of genetic signals as the percent of total sequences (y-axis) recovered for species in Treatments 1–5 (x –axis; T1, n = 1; T2–T5, n_replicate_ = 4; n_total_ = 17) constructed with equal proportions of biomass per non-target species (T1–T5) and spiked with decreasing amounts of target (*P. semilunaris*) tissue (T2–T5). The genetic signal for the target taxon was observed in two replicates in T3, one replicate in T5 and was not present in T4 replicates.

DNA based biodiversity estimates in treatments with ≤0.1% target biomass (T1, T3, T4, T5; Fig. 1) did not correspond to constructed biodiversity. Moreover, considerable variation between genetic signal strength and corresponding biomass proportion was observed for some non-targets in the same treatments with lower relative abundance of target biomass (Fig. 1). Most notably, the signal for non-target *N. hudsonius* represented a much larger proportion of sequences (52.73%) than biomass (24.96%) and greatly outnumbered sequences recovered for the other non-targets *E. lucius, G. aculeatus* and *A. rupestris* (Fig. 1). Disparity was also observed in T2 replicates constructed with 20% biomass from each species (Fig. 1), where target A represented 4.30% of total sequences, and *N. hudsonius* again made up a disproportionately large percentage (43.5%) of the total sequences.

In total, 2.9% of Trial A sequences (combined sequencing runs) with expected taxonomic classification fell below filtering thresholds set to identify potential false presences. After filtering, signal for target A was reduced by 0.03% (40 sequences) in T2 and detectability did not change. In replicates with target biomass equal to 0.1% (T3) and 0.02% (T5) of total sample mass, target signal fell below filtering threshold values and was not detected in these treatments after filtering. Detection of non-target species did not change after filtering and relative signal strength for non-target species varied from pre-filtered signals by ±1% per species.

### Analysis of COI sequence biodiversity in constructed samples with varying degrees of species richness (Trial B)

Trial B was designed to compare the limits to detection between samples constructed with varying degrees of species richness (S) to simulate a portion of the inherent variation observed in natural community samples. Ten of the eleven taxa used to construct test mixes were positively detected in corresponding individual controls. Initially, genetic signal for non-target *M. salmoides* was not detected in its individual control and was only observed in 1 of 22 samples constructed with *M. salmoides* tissue. We re-assigned taxonomy to OTUs initially classified as ‘Perciformes spp.’ using the GenBank^48^ which resulted in signal amplification and positive detection of non-target *M. salmoides*. All reported results are from data after re-assigning taxonomy for *M. salmoides*.

**Figure 2:**
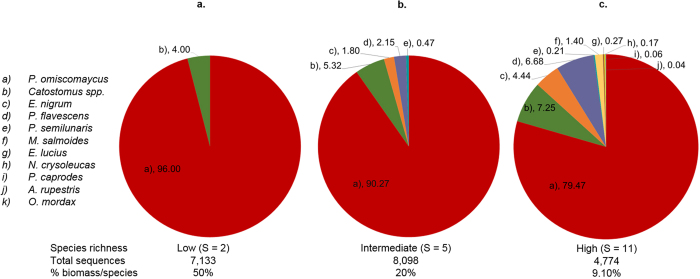
Distribution of genetic signals as the percent of total sequences recovered for each taxon (pie chart values) obtained from metabarcoded larval fish tissue samples constructed for Treatment 2 (T2) in Trial B subsets before setting false presence filtering thresholds. Panel (a) Low richness subset (n_replicate_ = 4). Panel (b) Intermediate richness subset (n_replicate_ = 4). Panel (c) High richness subset (n_replicate_ = 4). T2 replicates were constructed with equal proportions of biomass between the target (*P. omiscomaycus*) and all non-target species in each particular subset.

**Figure 3:**
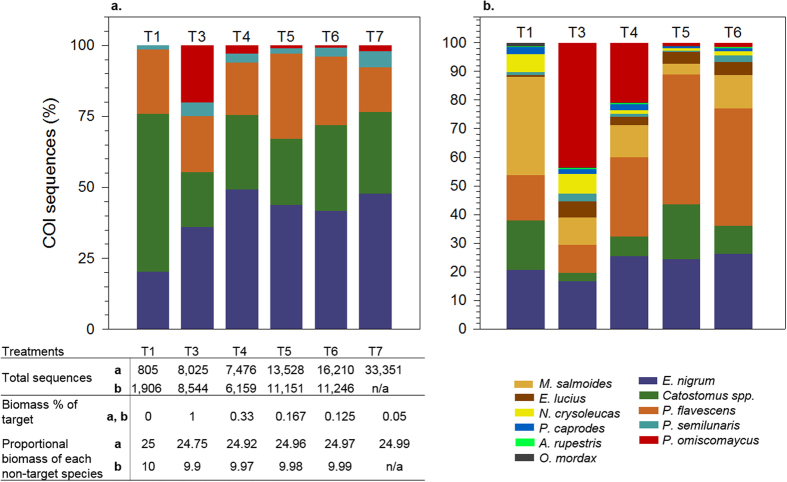
Metabarcoding results from larval fish tissue samples constructed for Trial B subsets before setting false presence filtering thresholds. Panel (a) Intermediate richness subset (S = 5). Panel (b) High richness subset (S = 11). The genetic signal distribution as the percent of total sequences recovered for species in each treatment (T1, n_replicate_ = 2, T3–T7, n_replicate_ = 4; S = 5, n_total_ = 22; S = 11, n_total_ = 18) constructed with equal proportions of biomass per non-target species (T1, T3–T7) and spiked with decreasing amounts of target (*P. omiscomaycus*) tissue (T3–T7).

In total, there were 270,053 COI sequences after filtering for quality and error of which 99.7% received the expected taxonomic classification. Average percent base similarity of our sequences to reference sequences used to assign taxonomy was 96.8%. Genetic signal for target B, *P. omiscomaycus*, was detected in all treatment replicates spiked with target tissue in each richness subset constructed to simulate low (S = 2), intermediate (S = 5), and high (S = 11) species richness. Genetic signals and associated biomass percentage for each taxon were markedly dissimilar in replicates constructed with proportionate biomass between all taxa in each richness subset (Fig. 2). Genetic signal for target B was considerably over-represented, with sequence percentages approximately 2X, 5X and 9X greater than corresponding biomass percentages when richness was low, intermediate, and high, respectively (Fig. 2). Some non-target genetic signals were also substantially skewed from corresponding biomass percentages in intermediate and high richness subsets where target biomass was ≤1% (Fig. 3).

Genetic signals for all non-target species were detected in all treatment replicates constructed with low and intermediate richness. In contrast, COI sequences were routinely recovered in all treatment replicates for only four of ten non-target species in treatment replicates constructed with high richness. The remaining non-target species were not detected in 36–95% of treatment replicates and 14 of the 26 total false absences occurred in T2 replicates constructed with equal amounts of biomass from target and non-target taxa.

In total, 0.51% of sequences with expected taxonomic classification fell below filtering thresholds set to identify potential false presences. In contrast to target A, the additional filtering did not affect the lowest limit of detection for target B. After filtering, non-target relative signal strength varied only slightly from pre-filtered signals (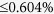) and detection rates for non-target signals did not change in treatments constructed with low and intermediate richness. In treatments with high richness, filtering resulted in signal loss and reduced detection rates for some non-target species; the overall occurrence of false absences associated with non-target species increased by 5% and the largest error rate increase (10%) occurred in T2.

## Discussion

High throughput metabarcoding methods (HTS) have the capacity to provide a practical, and quicker alternative to traditional morphological identification^25^,^26^,^27^,^30^, but we must understand the associated detection limits before incorporating HTS into an early detection monitoring program. In principle, the failure to recover a genetic signal from a species known to be present in our experiments, by design, provides information about the limits to detection in metabarcoded samples. The main findings from our assessment of detection sensitivity and accuracy associated with metabarcoding experimentally constructed larval fish assemblages are that we can detect species with biomass percentages as low as 0.02% of total sample mass, but that detection limits varied interspecifically, and in some cases sequence ratios were considerably different from the corresponding biomass ratios. The signal observed for *P. semilunaris* was under-represented relative to other species when all were present with biomass of equal proportions in Trial A, T2 (Fig. 1) and also in all Trial B treatments constructed for subsets with intermediate richness (Figs 2b and 3a) and high richness (Figs 2c and 3b). In contrast, the signal for target B, *P. omiscomaycus*, was consistently over-represented relative to constructed biomass percentages and despite increased matrix complexity, we detected target B in all treatments. In this case, detection of rare biomass was likely improved by the favorable PCR bias exhibited toward target B. Furthermore, despite having an equal or greater biomass proportion relative to *P. omiscomaycus*, we were unable to recover signals for six of the ten non-target species in Trial B subset with high species richness, and when we did detect them, their signals were usually represented by very few sequences.

Although species composition was accurately determined for many metabarcoded samples, our ability to detect a species was impaired by factors that skewed genetic signal from corresponding biomass abundance. In some cases, the bias caused false absences, and thus an increase in non-detection. Although sequence data filtering methods aimed at eliminating biologically irrelevant sequences and reference sequence database completeness (e.g., the *M. salmoides* case mentioned earlier) contributed to this skew, differential COI amplification (PCR bias) expressed by each specific mix of taxa had the largest influence on detectability. Our results from Trial B suggest that increasing sample complexity by adding more species did not impede our ability to detect species that are rare in terms of biomass; however, because the degree of bias expressed by a species depends on the mix of species present (as our Trial B results show), we cannot generalize how bias impacts measures of species richness. Comparisons between Trial A and B results suggest the limits to detection vary interspecifically because PCR bias increased the risk of non-detection for some taxa. Therefore, instrument sensitivity to rare biomass may be understated and results for the lowest limits of detection, while valid for our specific experiments, are not absolute for all sample mixtures. Instead, sensitivity and accuracy associated with metabarcoding will likely vary with species composition.

The accuracy of biodiversity measurements derived from metabarcoded samples can be improved if COI amplification bias is predictable or reducible. The primary source(s) of bias must be understood to determine if bias can be predicted and whether or not changes to the metabarcoding workflow can reduce the potential for biases to be expressed. Common sources of bias include PCR drift, interspecific variation in gene copy number, denaturation efficiency, and primer binding affinity^31^,^32^,^33^. In larval fish communities, the extent of variation in mitochondrial densities is unknown, however, we assumed there was some degree of variation and tried to limit it by constructing samples from specimens in similar developmental stages. PCR errors, denaturation efficiency, and primer binding affinity are artifacts of PCR. As our experimental design tried to limit bias due to differential COI densities, the PCR program used in our study was designed to limit bias originating from other sources as well. We attempted to reduce bias resulting from random amplification, a minimal contributor to bias by pooling multiple PCR replicates. Additionally, we could have reduced the total number of PCR cycles to limit differences in the copying rate^32^. Nonetheless, if PCR errors were the sole cause of bias in our samples, the similar signal skew observed across replicates would not have occurred. To limit bias associated with differential denaturation efficiency, we included a reduced annealing temperature, and low template to reaction volume ratio, as has been used for multi-templates containing a mix of AT and GC rich genes. Comparisons between sequencing results from samples amplified using a single primer^49^ and the primer combination used in our study^45^ revealed intraspecific differences in signal strength and detection error rates. The amplification bias observed in our study was likely caused by a species-specific response to the primer design.

Although HTS studies focused on diverse marine and freshwater invertebrate taxa^28^,^29^,^30^,^50^ have demonstrated barcode and primer selection influence the accuracy of species richness estimates in metabarcoded samples^24^, this effect has not been previously documented in larval fish assemblages. The COI barcode was the preferred option because species-level identifications^18^,^51^,^52^ and an existing reference database^47^ are essential elements to improve species detection probabilities using HTS for a non-targeted AIS early detection strategy. Nonetheless, the extreme amplification biases resulting in non-detection of some species in our study demonstrate the drawbacks of using COI for species detection. Reducing the effects of bias and improving species detection probabilities in metabarcoded samples, however, would also be possible in conjunction with other mitochondrial markers such as cytochrome b (cyt b), 12S rRNA, or 16S rRNA with multiple primers^53^. An additional advantage to using multiple markers is that measures of biodiversity can be compared across markers as a means of cross-verification of the taxa detected per sample^54^.

While differential amplification of the COI barcode considerably influenced detection error rates, we identified other areas within the HTS workflow that also need refining to reduce non-detection risk. From the initial results from Trial B (S = 11) we learned that reference sequence databases used to assign taxonomy to unknown barcode sequences can contain flaws including low resolution taxonomic classification or taxonomic synonyms and misspelled names that can cause detection errors. Therefore, the ability to detect is limited by the reference database used to assign taxonomy. Unlike PCR bias, detection errors associated with this factor are easily corrected and libraries used in early detection assays should be revised to eliminate such flaws. In addition, sample collection, handling, processing and preservation methods should minimize the chance for DNA degradation and contamination to produce samples that yield high quality DNA. Currently, we have the tools and knowledge for the effective collection and preservation of larval fish samples but, we must ensure these methods are implemented in the field and lab. Methods used in downstream processes such as the metabarcoding PTP layout, and parameter settings selected for bioinformatics processing can also affect the accuracy of sequencing results^35^. In our study, under-represented signals usually fell below filtering thresholds set to identify and isolate false presences. Similar methods used to define signal strength gradients and identify false presences in natural community samples with unknown biodiversity may be increasing the likelihood of non-detection of rare or negatively biased species. Recent studies confer additional support for this conclusion^30^,^31^,^32^,^33^,^34^,^35^, but a generalized approach to handling under-represented signals in the context of the rare species detection has yet to be developed.

Our study provides insight into the limits to detection associated with metabarcoding analysis of larval fish communities. This approach was sensitive enough to detect the presence of species with biomass as low as 0.02% and 0.05% of total sample mass for target A, *P. semilunaris* and target B, *P. omiscomaycus*, respectively. The observed lowest limits of detection are far from theoretical expectations based on the possible instrument plating layouts and corresponding estimated number of output sequences per sample. Given that the lowest theoretical detection limits were not tested in our study we may be able to detect species rarer than we reported. Moreover, our results indicated that PCR bias can skew genetic signals and increase non-detection risk, therefore, the limits to detection seem to be specific to each species and may vary with sample composition. However, detection limits may become more uniform between species if using better designed primers and multiple genetic markers to smooth the effects of PCR bias.

Though PCR bias and other challenges remain in the development of HTS for biomonitoring, the potential benefits of these molecular methods warrant continued investigation and experimentation to solve these problems. Incorporation of improved PCR primers, primer cocktails, and the use of multiple genetic markers has a strong chance of removing many PCR bias issues. Beyond improvements in lab techniques, the field of HTS is rapidly changing and growing, providing greater opportunities to test detection limits through increased depth of coverage and more accurate DNA sequencing chemistries. Databases of genetic information grow more quickly each year, thereby increasing the available genomic data for further development of new markers and new methods of analyzing metagenomic data sets. The field of environmental genomics is still quite young, but continues to create new avenues for applying genetic information to biomonitoring as long as methods are properly vetted and tested for environmental applications.

## Additional Information

**How to cite this article:** Hatzenbuhler, C. *et al*. Sensitivity and accuracy of high-throughput metabarcoding methods for early detection of invasive fish species. *Sci. Rep.* **7**, 46393; doi: 10.1038/srep46393 (2017).

**Publisher's note:** Springer Nature remains neutral with regard to jurisdictional claims in published maps and institutional affiliations.

## Figures and Tables

**Table 1:** Summary of experimental design for Trial A.

Treatment	Target	Non-target			
	*Proterorhinus semilunaris*	*Notropis hudsonius*	*Ambloplites rupestris*	*Esox lucius*	*Gasterosteus aculeatus*
T1	n/a	25.00	25.00	25.00	25.00
T2	20.00	20.00	20.00	20.00	20.00
T3	0.10	24.98	24.98	24.98	24.98
T4	0.04	24.99	24.99	24.99	24.99
T5	0.02	24.995	24.995	24.995	24.995

Tissue homogenates for each species were mixed prior to DNA extractions to achieve the following biomass ratios. Approximate relative biomass abundance per taxon as a percent of total sample biomass for single species controls (not listed) and treatment replicates (single species control, n = 1 per species; T1, n_replicate_ = 1; T2–T5 n_replicate_ = 4; n_total_ = 22). Common names for taxa from left to right; Tubenose Goby, Spottail Shiner, Rock Bass, Northern Pike, Three Spine Stickleback.

**Table 2:** Summary of experimental design.

Richness treatment	Target	Non-target									
	*Percopsis omiscomaycus*	*Catostomus* spp.	*Perca flavescens*	*Etheostoma nigrum*	*Proterorhinus semilunaris*	*Micropterus salmoides*	*Ambloplites rupestris*	*Percina caprodes*	*Osmerus mordax*	*Notemigonus crysoleucas*	*Esox lucius*
**Low**											
T1	n/a	n/a									
T2	50	50									
T3	1	99									
T4	0.33	99.67									
T5	0.167	99.83									
T6	0.125	99.88									
**Intermediate**											
T1	n/a	25	25	25	25						
T2	20	20	20	20	20						
T3	1	24.75	24.75	24.75	24.75						
T4	0.33	24.92	24.92	24.92	24.92						
T5	0.167	24.96	24.96	24.96	24.96						
T6	0.125	24.97	24.97	24.97	24.97						
T7	0.05	24.99	24.99	24.99	24.99						
**High**											
T1	n/a	10	10	10	10	10	10	10	10	10	10
T2	9.09	9.09	9.09	9.09	9.09	9.09	9.09	9.09	9.09	9.09	9.09
T3	1	9.9	9.9	9.9	9.9	9.9	9.9	9.9	9.9	9.9	9.9
T4	0.33	9.97	9.97	9.97	9.97	9.97	9.97	9.97	9.97	9.97	9.97
T5	0.167	9.98	9.98	9.98	9.98	9.98	9.98	9.98	9.98	9.98	9.98
T6	0.125	9.99	9.99	9.99	9.99	9.99	9.99	9.99	9.99	9.99	9.99

Trial B richness (S) subsets with low (S = 2) intermediate (S = 5) and high (S = 11) species richness. Approximate relative biomass abundance per taxon as a percent of total sample biomass for single species controls (not listed) and treatment replicates (single species control, n = 1 per species; T1, n_replicate_ = 2; T2–T7 n_replicate_ = 4; n_total_ = 79). Common names for taxa from left to right; Troutperch, White & Longnose Suckers, Yellow Perch, Johnny Darter, Tubenose Goby, Largemouth Bass, Rock Bass, Logperch, Rainbow Smelt, Golden Shiner, Northern Pike.

**Table 3:**
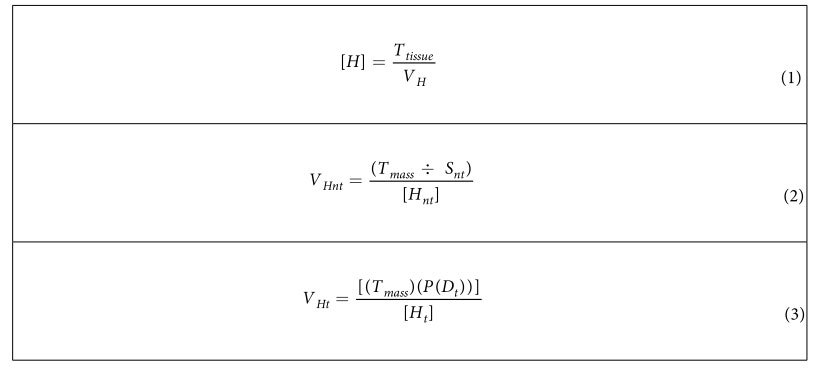
Equations (eq.) used to calculate (eq. 1) tissue homogenate (*H*, 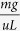 ) concentrations and (eq. 2, 3) homogenate volumes (*V*_*H*,_ μL) for non-target (*nt*) and *t*arget (*t*), respectively.

For eq. 1, *T*_*tissue*_ is the total mass of cryogenic tissue homogenate used from a single species. For eq. 2 total sample mass (*T*_*mass*_) and number of non-target taxa (*S*_*nt*_) and for eq. 3 (*T*_*mass*_) and probability of detection for target species *P(D*_*t*_) at the corresponding ratio of target mass to total sample mass.
